# Organization of GC/MS and LC/MS metabolomics data into chemical libraries

**DOI:** 10.1186/1758-2946-2-9

**Published:** 2010-10-18

**Authors:** Corey D DeHaven, Anne M Evans, Hongping Dai, Kay A Lawton

**Affiliations:** 1 Metabolon, Inc., 800 Capitola Drive, Suite 1, Durham, NC 27713, USA

## Abstract

**Background:**

Metabolomics experiments involve generating and comparing small molecule (metabolite) profiles from complex mixture samples to identify those metabolites that are modulated in altered states (e.g., disease, drug treatment, toxin exposure). One non-targeted metabolomics approach attempts to identify and interrogate all small molecules in a sample using GC or LC separation followed by MS or MS^n ^detection. Analysis of the resulting large, multifaceted data sets to rapidly and accurately identify the metabolites is a challenging task that relies on the availability of chemical libraries of metabolite spectral signatures. A method for analyzing spectrometry data to identify and **Qu**antify **I**ndividual **C**omponents in a **S**ample, (QUICS), enables generation of chemical library entries from known standards and, importantly, from unknown metabolites present in experimental samples but without a corresponding library entry. This method accounts for all ions in a sample spectrum, performs library matches, and allows review of the data to quality check library entries. The QUICS method identifies ions related to any given metabolite by correlating ion data across the complete set of experimental samples, thus revealing subtle spectral trends that may not be evident when viewing individual samples and are likely to be indicative of the presence of one or more otherwise obscured metabolites.

**Results:**

LC-MS/MS or GC-MS data from 33 liver samples were analyzed simultaneously which exploited the inherent biological diversity of the samples and the largely non-covariant chemical nature of the metabolites when viewed over multiple samples. Ions were partitioned by both retention time (RT) and covariance which grouped ions from a single common underlying metabolite. This approach benefitted from using mass, time and intensity data in aggregate over the entire sample set to reject outliers and noise thereby producing higher quality chemical identities. The aggregated data was matched to reference chemical libraries to aid in identifying the ion set as a known metabolite or as a new unknown biochemical to be added to the library.

**Conclusion:**

The QUICS methodology enabled rapid, in-depth evaluation of all possible metabolites (known and unknown) within a set of samples to identify the metabolites and, for those that did not have an entry in the reference library, to create a library entry to identify that metabolite in future studies.

## Background

Metabolomics is the study of the small molecules (i.e., metabolites or biochemicals), contained in a cell, tissue, organ or biological fluid [1-3]. Metabolomics data can be generated from an array of sources such as liquid or gas chromatography coupled to mass spectrometry (e.g., LC/MS, GC/MS), capillary electrophoresis (CE), and nuclear magnetic resonance (NMR) spectroscopy [4]. Typically, metabolomics uses non-targeted methods where the analytical conditions are optimized to detect and identify as many molecules as possible. However, targeted metabolomics methods where the chromatography is optimized for detection of a specific molecule or class of molecules (e.g., lipids) are also used. In either case, the structure of metabolomics data is generally three dimensional. For example, the data for a separation method coupled with mass spectrometry includes values for time, intensity and mass (m/z).

The fundamental goal of metabolomics analysis is to quickly and accurately identify the metabolites detected in a complex biological sample and determine which change (increase or decrease) in response to experimental conditions (e.g., disease state, drug treatment, etc). Typically, data for a set of biological samples are collected, plotted and stored in individual files with each file corresponding to each biological sample. Using various software tools, the raw three-dimensional data for the sample set are integrated into ion peaks organized by mass, retention time (RT), and peak area. The integrated ion peaks are aligned by time and may be normalized by intensity across the set of samples. Then, each individual sample is processed for the identification of metabolites which, in most cases, involves the comparison of individual spectra to standard reference libraries. Such standard reference library data consist of known spectra corresponding to certain metabolites that may be present in a given sample. While individual ions may be detectable in such spectra, the combinations and interplay of such ions to indicate specific individual metabolites may not be immediately discernable, especially in only a single biological sample. If the individual sample contains substantially pure components (such as small molecule metabolites), the spectrum of the component can be easily matched with the spectra of known metabolites in order to identify the biochemical. However, in many cases, the fractionation of a particular biological sample (in a liquid or gas chromatograph, for example) is incomplete. In this case, two or more biochemicals may co-elute from the incomplete chromatographic separation process giving rise to an impure mixture of metabolites going into the spectrometer. The conventional methods of analyzing datasets by grouping and organizing related ions on a per sample basis fall short when faced with this level of data complexity [5-7].

An analytical method that is capable of performing statistical analysis on a set of ions in a given population (sample set) could address these shortcomings [7]. Recently, a correlation-based deconvolution approach was reported for LC/MS datasets [8]. In this report, we present QUICS, a method to identify and organize the ions related to metabolites of known and unknown identity from complex mixtures. The function of QUICS is to go beyond a single sample approach to the identification of the multiple ions that are related to any given metabolite by correlating ion data across a set of samples. Consequently, when viewed over many individual biological samples of the same type, subtle spectral trends indicative of the presence of one or more otherwise obscured metabolites may be revealed. Once these related ions are grouped based upon the correlation across samples, there exists the capability of searching for these organized ion groups in reference library databases to identify the corresponding metabolites. Furthermore, new library entries can be created when grouped ions represent a new, undocumented metabolite (unknown). Here, we show the utility of this method for the deconvolution and analysis of GC-MS and LC-MS^n ^data sets.

## Results and Discussion

**Figure 1:**
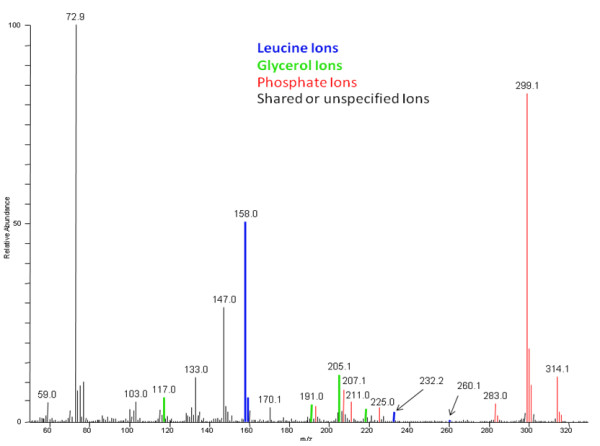
**A single scan from an EI GC/MS analysis of a liver biopsy**.

**Figure 2:**
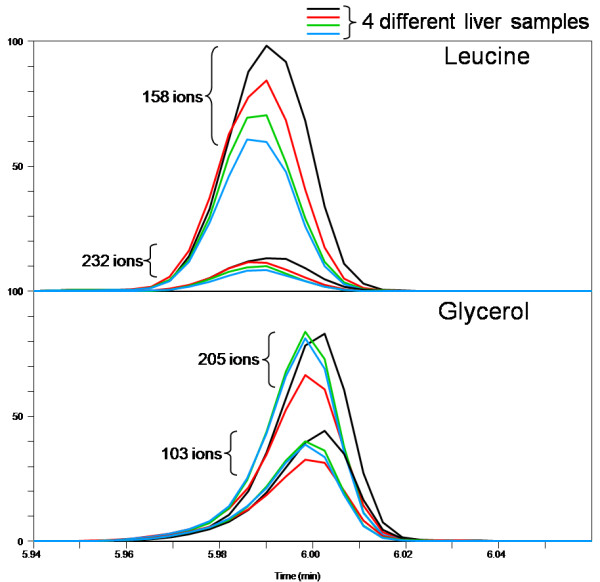
**The selected ion chromatogram (SIC) for two different ions from leucine (A) and two ions from glycerol (B) as measured from 4 different liver sample analyses**. The ions from leucine, 158 and isotope 232 m/z, trend across the different liver samples and the ions related to glycerol share a different trend.

**Figure 3:**
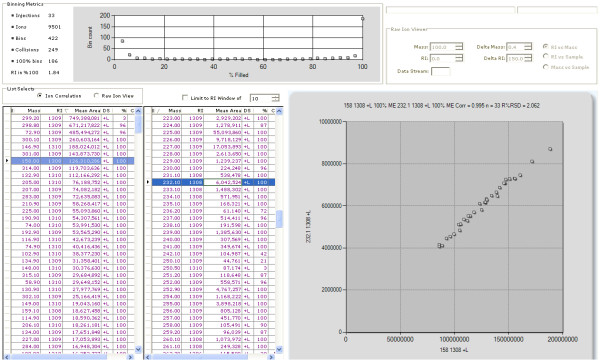
**Correlation of m/z 158 and 232, two ions related to leucine, across all injections in a study**. All ions were pulled from all sample injections in a study and analyzed all for correlation. Ions are then grouped based on a user-specified correlation limit.

**Figure 4:**
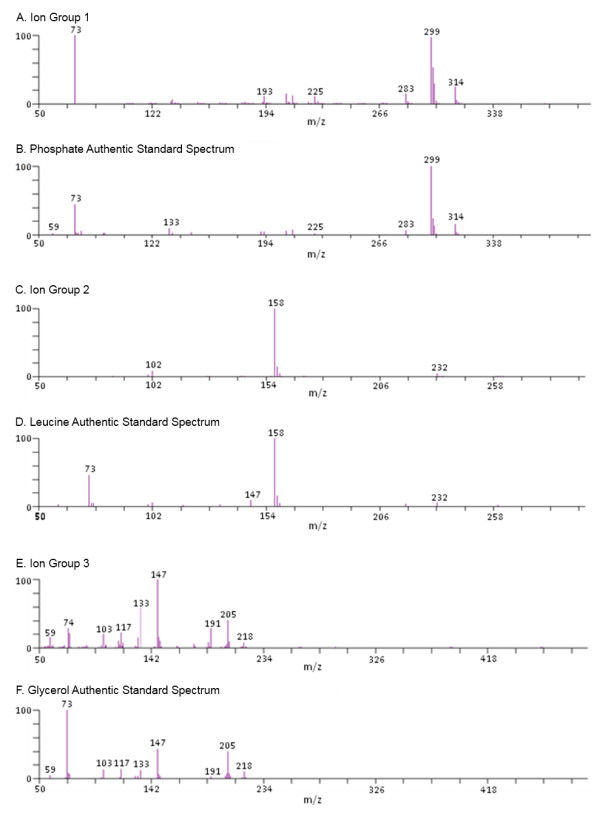
**The creation of 3 different groups of correlating ions, A,C, and E, and their respective authentic standard library entries are shown for comparison, B, D, and F**. The ions within the groups correlated with a minimum of 0.8. From the single scan in Figure 1, 3 different compounds are present; phosphate, leucine and glycerol.

To demonstrate the ability of the QUICS method to accurately separate and organize ions related to co-eluting biochemicals, a sample set of 33 liver samples was analyzed by GC/EI-MS. In this study three known metabolites, leucine, phosphate and glycerol, consistently co-elute as shown in Figure 1. Without prior knowledge of the content of this scan it would be difficult to determine whether the spectrum shown in Figure 1 is a mixture of metabolites or a single metabolite. Using the QUICS methodology this spectrum is separated into its three biochemical components by grouping highly correlated individual ions based on instrument response across the sample set. This is possible because the ions originating from a single biochemical will exhibit similar biological variability across the study and therefore correlate, as can be seen in Figure 2. Consequently, individual ions belonging to a single component can be grouped based on correlation. In Figure 2, Panel A demonstrates the chromatographic profile of two ions generated during a GC/EI-MS analysis of leucine, 158 and 232 m/z. Note that the intensities of the ions trend the same way across the four different liver samples shown. More specifically, the liver sample shown in black has the highest amount of 158 and 232 m/z, followed by the liver sample represented in red, then green, then blue. As a result, these two ions correlate when the ion response is compared across all of the sample injections in the study which is shown in Figure 3. This is in contrast to the ions related to glycerol (Figure 2, panel B), in which the liver sample labelled in black has the highest amount of both ions (205 and 103 m/z), followed by green, then blue, then red. The three ion groups that were created from a set of 33 liver samples and their respective authentic standard spectrum matches are shown in Figure 4.

**Figure 5:**
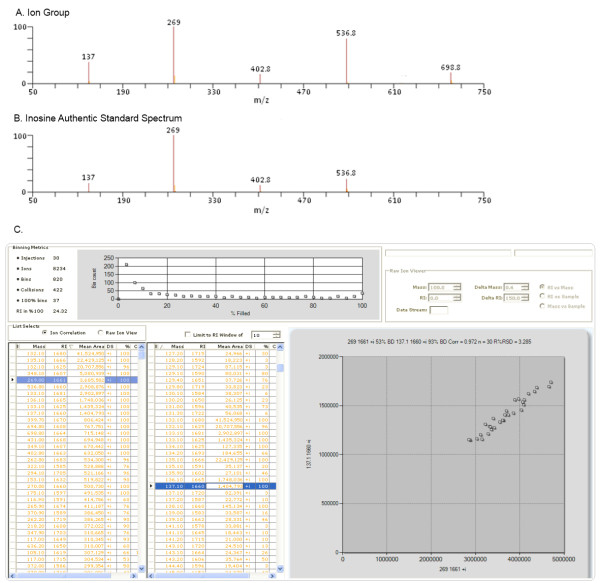
**(A) An ion group including the protonated molecular ion, isotopes, adducts, and multimers of inosine (m + H^+ ^269) based on correlation across a 33 sample set study**. (**B**) The authentic standard spectrum of inosine. (**C**) The correlation between the protonated molecular ion of inosine at 269 m/z and an in-source fragment at 137 m/z.

The examples discussed thus far are from data generated from electron ionization (EI) GC/MS analyses, where all of the ions detected are a result of fragmentation of the intact molecule during ionization. However, the QUICS method is also useful with other types of data. Deconvoluting LC/MS data where a biochemical compound does not necessarily fragment in the source but instead readily forms adducts and multimers is also possible. Shown in Figure 5, Panel A is an example of ions from an LC/MS sample injection that were grouped based on correlation. The ions presented are various adducts, isotopes, multimers and in-source fragments for inosine, as confirmed by the authentic standard library spectrum in Panel B. Shown in Panel C is the correlation between the protonated molecular ion at 269 m/z with the in-source fragment at 137 m/z across the sample set. The QUICS method is also applicable to LC/MS applications where all ions eluting from the chromatographic system are fragmented rather than individual masses being isolated and then fragmented. In this case, the fragment ions from the individual metabolites will also correlate depending on biological variability.

The ability the QUICS method to separate co-eluting species is dependent upon the correlation of the detected ion area responses across an entire study. One potential complication to this type of analysis is that ions from compounds that have limited biological variability across the sample set will not be highly correlated because there is limited variation in ion signals. In addition, correlation calculations can be confounded by ions that are shared among the co-eluting compounds. In these cases, the ion response is the sum of all the co-eluting compounds and therefore correlation might be compromised. An example of this phenomenon is the 147 and 72.9 m/z ions shown in Figure 1. These ions are common to all three co-eluting compounds as can be seen in the authentic standard spectra in Figure 4, panels B, D, and F. Ultimately these ions were grouped with the compound that had a sufficient degree of correlation, specifically 73 with phosphate (Figure 4, panel A) and 147 with glycerol (Figure 4, panel E); neither ion correlated sufficiently to be grouped with other leucine ions (Figure 4, panel C) even though the leucine standard also produced these ions (Figure 4, panel D). While these potential complications do exist they rarely interfere to a significant enough extent to compromise the quality of the generated spectrum.

**Figure 6:**
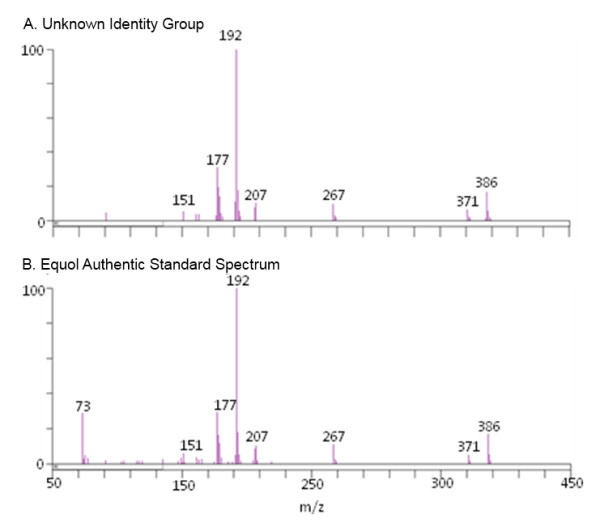
**(A) An ion group created from an EI-GC/MS analysis of urine that when created was an unknown**. (**B**) The authentic standard spectrum of equol, later permitted the identification of the unknown.

While the example in Figure 4 focuses on a region of chromatography where three known compounds co-elute, the QUICS method is most powerful when the chemical composition of a sample set is unknown. In that instance, individual ions originating from each individual chemical can be grouped even when the chemical identity is unknown and possibly co-eluting with other unknowns. Presented in Figure 6, Panel A is an example of a spectrum that was created using the QUICS method. The ions that were grouped (Figure 6, panel A) as originating from an unknown source were later identified as Equol (4',7-isoflavandiol), an isoflavandiol metabolized from daidzein, a type of isoflavone, by bacterial flora in the intestines [9] (Figure 6, panel B).

The ultimate goal of the QUICS method is to permit the deconvolution of the many redundant ion signals that each individual biochemical entity produces. One of the standard approaches of data analysis in the biochemical profiling field is to perform statistical analyses using every individual ion signal whether or not they are redundant ions produced by a single chemical [10-12]. This ion-centric approach leads to a greater number of false discoveries as a result of increased numbers of measurements processed in the statistical analysis. This approach also has the potential to skew statistics since different chemicals will produce different numbers of ion signals. For example, multivariate techniques such as Principal Component Analysis (PCA) can be skewed in favor of chemicals that produce more ion signals. In contrast, the QUICS method enables a chemo-centric approach to data analysis. Once the related ion features that belong to a given biochemical are organized and grouped, a single ion from that group can be used to represent that metabolite in statistical analyses. By using the chemo-centric approach the number of false discoveries is reduced since the number of ions processed in statistical analyses is reduced to a single representative ion for each metabolite and, furthermore, the potential for skewed statistical results is decreased.

Similar use of correlation analysis across samples has been used with NMR data. In that analysis, the multiple chemical shift peaks generated by a single molecule can be correlated across samples, grouped, and used to aid identification of detected molecules [13-16]. While similar in concept to the method presented here, the ultimate outcome has different advantages since the underlying data streams are so unique. One of the goals of the QUICS method was to deconvolute the highly redundant ion features generated by a single molecule traditionally seen in most mass spectrometric based technologies. As discussed, this redundancy of data can alter statistical analysis and lead to greater numbers of false discoveries. Using the QUICS method, it is possible to group all of the redundant ions related to the detected metabolites within a set of experimental samples with or without the use of a known spectral library. By utilizing both the chromatographic time domain and ion response the QUICS method is able to gain an additional degree of specificity not seen in NMR data streams. As a result only those ions that co-elute and correlate are grouped, thus removing the confounding redundancies. It should be noted that this method represents an automated package that also enables the generation of spectral library entries for unnamed/unknown metabolites--those molecules where a reference library entry of chemical spectral signatures does not yet exist. Taking a statistical approach to the analysis of all ions in a sample and evaluating the entire sample set simultaneously enables the system to not only identify metabolites with spectral matches to known biochemicals in the reference library, but also enables identification of biochemicals that are not in the reference library. The identification of these so-called unnamed metabolites capitalizes on the fact that ions originating from a single biochemical will exhibit similar biological variability across the study samples and therefore correlate. The QUICS method has been used successfully for diverse experimental studies including disease biomarker identification, drug mode of action, toxicology, aging and characterization of variation in complex mixtures such as milk and on a variety of sample matrices (e.g., biological fluids, tissue, milk) [17-22].

### Experimental

To begin, GC/MS and LC/MSMS data are collected as part of an automated, high-throughput processing system. These data consist of ions peaks that have been automatically detected and integrated from raw 3D GC/MS or LC/MSMS analyses for each sample. These data are characterized by the mass (m/z) of the ion, the area (representing the amount of the ions), and retention time (RT) and retention index (RI) representing the chromatographic characteristics that tell when the related biochemicals elute. Retention indices are assigned to all ion peaks by calibration with the retention index of internal standards, added into each sample, and their retention time for each sample [6]. These raw signal data and integrated ion peak data are both loaded and stored in a relational database system which provides data structures that are optimal for the storage and retrieval of large chromatographic datasets. Immediately after data acquisition has been completed for a sample, that data file has been retrieved by the system, and automated loading and peak detection have been completed, the resulting data is compared using a matching algorithm against an existing spectral library which contains the spectral definitions of both known standards as well as unknown metabolites. Any matches between ion peak data and the spectral library that occur are scored for confidence, and that information is stored within the relational database system.

To determine which groups of ions are common across samples, all the ions are binned by mass window and RI window. Ions from the same chemical that are determined to be common across samples are then grouped by correlation. Table 1 shows the processing thresholds for ion binning and bin grouping.

**Table 1:** Binning and Grouping Threshold Parameters

Name	Description	Normal Values
***Ions to Analyze***		

RI +/-	Range of retention indexes of ions across samples for analysis	0~9000 +/- 100

***Ion Binning***		

Mass Window	Mass window for ions to be binned together	0.4

Extended Mass Window	Mass window to look for ions missing from samples in neighboring bins.	0.5

RI Window	RI window for ions to be binned together	25 to 50, default 25

Extended RI Window	RI window to look for ions missing from samples in neighboring bins.	25 to 50, default 38

***Bin Grouping***		

Max. RI Difference	Maximal difference of the average RIs of the two bins	0~999

Min. Correlated Ions	Minimal number of common singlet ions existing in the two bins	1~999

Max. Linear Area	Maximal area of ions that can be included for correlation calculation	Large enough to include all ions

Max. RSD (%)	Maximal Relative Standard Deviation (%) of common singlet peak area ratio between the two bins	0~100

Min. Correlation	Minimal correlation value for grouping	0 ~ 1 with default of 0.8

### Ion Bin

The first step is to begin a binning process where ions from samples in the same study set are binned by mass and retention index (RI)[23]. A bin is a space partitioning data structure that enables fast region queries and nearest neighbor search. Each bin is characterized by a center mass and a center RI. Ions from across the set of samples are put into the same bin if their masses and RIs fall into the windows around the center mass and the center RI.

Two kinds of ions can be defined in this process, a singlet and a multiplet. A singlet ion comes from a sample that has one and only one ion in the same bin. A multiplet ion comes from a sample that has more than one ion in the same bin. Multiplet ions from the same sample imply the co-elution of several compounds and are termed a collision. The number of collisions, i.e. the number of times multiple ions were detected in all bins, seen in a loaded data set is displayed in Figure 5(C).

Binning includes the following steps:

1. Sorting ions by their areas in descending order.

2. Bin ions with smaller areas around ions with larger areas, with the larger ions serving as the bin centers.

3. Calculate the statistics of each ion bin: mean mass, mean area, mean RI and their standard deviations, respectively, from all singlet ions in the bin.

4. Reset the bin center mass and center RI to its mean mass and mean RI to take into account the ion distribution within the bin. Remove all bins that have no singlet ions.

5. Re-binning all ions into these bins. If an ion can't be binned into any of them, a new bin is created with its mass and RI as the center mass and RI.

6. Repeat steps 3 and 4 for optimized binning of all ions.

The number of singlet ions in an ion bin indicates how popular this ion is across the samples. The size of a bin is represented by the percentage of singlet ions among the total number of samples, the percentage of "filled" in Figure 5 (C, % column).

When a bin is not 100% filled, that is, when there are samples that have no ions in the bin, it might be possible that these samples might have the same ions but they might be just outside the mass window and/or the RI window of the bin. Should these outlier ions be the same as those in the bin, their areas would be within four standard deviations of the areas of singlet ions in the bin. To recover these outlier ions from the missing samples, ions within an extended mass window and/or RI window are searched for those samples in the bins with lower "filled" percentage; if found, such ions are migrated from the less "filled" bin to the more "filled" bin.

For example, suppose there are 30 samples in total, and there are 25 samples that have singlet peaks in bin B1, and sample A does not have any peak in it. To check if sample A has an outlier peak that is similar to peaks from other samples in bin B1, peaks from sample A in neighbor bins are searched in an extended RI and mass window. Peaks from sample A must have their peak areas in the window of four standard deviations of peak areas in bin B1. The best matched peak from sample A in the neighbor bins would be migrated to bin B1, making bin B1 more "filled" (sample A is now included).

This process is looped from the more filled bins to the less filled bins.

### Grouping Ion Bins

In GC/MS or LC/MS, many ions may be produced from the same metabolite during the ionization and fragmentation processes. In LC/MS, different adducts and aggregates may form from the same metabolite. These ions from the same metabolite should be well correlated. On the other hand, ions from different chemical origin are largely non-covariant.

Once ions from across the set of samples are properly binned, each bin represents a common ion that is common in many samples in the sample set. Bins representing ions from the same metabolite should be well correlated when analyzed across multiple samples. Suppose a majority of the samples contain a common metabolite A, which has ionized to N ions, then there would be N bins that are well correlated. The goal of grouping bins is to find those well correlated ions that could represent a known or unknown metabolite in those samples.

### Correlation between the Normalization Bin and Correlation Bin

The Pearson's correlation is calculated to measure the correlation between two ion bins. Only singlet ions that are common in both ion bins are included in the calculation.

Bins are sorted by their mean area in descending order. Using the larger bins as the normalizer, smaller bins are grouped around the larger bins as if the correlation is above the correlation threshold value. The correlation between the normalizer bin and correlation bin is calculated as follows:(1)

where S_i _is the area of a common singlet ion,  is the average area of the common singlet ions in a bin. Thresholds for the minimal correlation and the number of singlet ions that are common in both the normalization bin and the correlation bin are user specified and can be altered prior to the process being started.

The correlation threshold is chosen by trial-and-error. Usually it is between 0.70 and 0.90. It depends on the matrix type and the sample set size (number of samples in the sample set). A too low correlation threshold would group too many bins into a group, whereas a too high threshold would miss some ions from the group, which can be judged from metabolites in the samples known from library match.

### Chemical Intelligence

The ions originating from the same chemical will have different m/z values and include various in-source fragmentations, isotopes, adducts and multimers. In LC/MS, ions could be aggregates of monomers or adducts with solvent/mobile phase ions such as H^+^, Na^+^, K^+^, Cl^-^, OH^-^, NH_4_^+^, H_2_O, COO^-^, etc. The true mass could be calculated from the measured mass:(2)

where N_IMER _is the number of monomers in the aggregate, (m/z)_adduct _is the (m/z) of the adduct, (m/z)_monomer_, is the (m/z) of the monomer, and (m/z)_measured _is the measured (m/z) for the ion.

Table 2 shows the most common aggregates and adducts. All ions in the group are checked against these possible aggregates and/or adducts to determine the most probable form of the ion.

**Table 2:** Possible aggregate and adducts in measured ions

**(m/z)**_**adduct**_	**N**_**IMER**_	Ion Form	PROBABILITY(%)
1.00728	3	3 m + H	1

1.00728	4	4 m + H	1

1.00728	5	5 m + H	1

22.98977	3	3 m + Na	1

22.98977	4	4 m + Na	1

22.98977	5	5 m + Na	1

1.00728	1	m + H	100

0	1	m-	10

1.00728	2	2 m + H	50

22.98977	1	m + Na	90

22.98977	2	2 m + Na	25

39.954	1	m + K	10

39.954	2	2 m + K	5

-1.00728	1	m-H	100

39.954	4	4 m + K	1

-1.00728	2	2 m-H	50

44.9971	1	m + Form	99

-18.01002	1	m-H2O	80

39.954	5	5 m + K	1

1.00728	3	3 m-H	1

-1.00728	4	4 m-H	1

-1.00728	5	5 m-H	1

20.9741	2	2 m + Na-2H	50

1.00728	6	6 m + H	1

1.00728	7	7 m + H	1

1.00728	8	8 m + H	1

-17.01	1	m + H-H20	1

34.9689	1	m + Cl35	12

36.9659	1	m + Cl35[Cl37]	4

18.03437	1	m + NH4	1

To calculate the monomer mass of a metabolite, each ion in the grouped bins is tested against the above possible ion form and the possible monomer mass is calculated and scored by the product of the ion peak area and the ion form probability.

To do so, possible monomer masses for all the ions in all ion forms are calculated and binned and scored in Table 3:

**Table 3:** List of possible monomer masses, binned and scored

Ion	Calculated Monomer Mass	Calculated Score	Assigned Bin
1	(m/z)_Mono_^1^	Score^1^	X

2	(m/z)_Mono_^2^	Score^2^	X

...	...	...	X

n	(m/z)_Mono_^n^	Score^n^	X

1. For ***each ion ***from big (large ion peak area)to small (small ion peak area) in the group AND ***each ion form ***from the most probable to less probable in the possible ion forms

○ Calculate the monomer mass (m/z)_Mono _and its score.

○ If some monomer masses have already been calculated AND this monomer mass is within one of them, add its score. e.g., if (m/z)_Mono_^i ^- 0.3 <=(m/z)_Monomer _<= (m/z)_Mono_^i ^+ 0.3, then SCORE_i _+ = (Peak Area) * (Ion Form Probability)

○ Otherwise, add this monomer mass and its score to the possible monomer mass list as another possible monomer mass.

2. Among the all possible monomer masses, the monomer mass with the maximal mass score is the most probable mass.

After the monomer mass is calculated from the well correlated bins in a group, other forms of adduct/aggregate ions from the same metabolite not existing in the bins in the group could be searched. These missing forms of adduct/aggregate ions could be more variant across samples and so their correlations with the normalize bin are below the threshold value used for grouping and so bins representing these adducts/aggregates are mis-grouped into different groups. The QUICS method attempts to correct these misgrouped ions by lowering the required correlation threshold for those ions which have masses consistent with the monomer mass of the metabolite.

To find the missing adduct/aggregate in a bigger group (with a larger peak area of normalize bin) with its monomer mass calculated as described above, for each possible form of adduct/aggregate:

1. Calculate the measured mass from the monomer mass assuming there is an ion in this form of adduct/aggregate.

2. Bins in smaller groups within are searched within the mass window (±0.4) of calculated mass and the RI window of the normalize bin of the larger group.

3. Calculate the correlation of these adduct/aggregate bins with the normalize bin of the big group.

4. The bin with the highest correlation above 0.4 will be one of the missing adduct/aggregate ions and will be migrated from the smaller groups to the big group.

This process is repeated for each group from big to small.

Isotope ions are checked the same way except they require the ion peak area to be no more than half of the normal ion.

As discussed above, each primary ion peak bin represents the average of a common ion across samples, and each group of correlated bins represents ions of a common metabolite. For LC/MS/MS, secondary MS2 ions for each primary ion are also retrieved from all the singlet samples in the primary ion peak bin and similarly binned. Among the MS2 ion bins that satisfy the minimal number of singlet ions, the maximal bin with the maximal mean intensity will be used as the normalize bin to normalize other bins that satisfy both the minimal number of singlet ions and the minimal relative intensity against the normalize bin, are included into the library to represent the MS2 ions for the primary ion. In summary, integrated primary ion chromatographic peaks from all the samples are binned based on chromatographic features of retention index and ion mass, well correlated bins within the retention index window are grouped to create a library entry to represent a chemical entity. Each mass with their characteristic values from the averages of mass, area, RI, and RT from a bin in the group, represents one of the fragmented ions from a pure chemical entity or its adducts/aggregate. For LC/MS/MS, secondary masses are also created from the bin/group of all secondary ions across the samples and the averages represent the secondary ions from one of the primary fragments of the chemical entity. Such created library entries for each chemical entity may match well to an entry in the library, or as an unknown to be added to the library and to be further identified with more studies.

### Reference Library Entries

Once the binned ions are grouped by correlation, the groups can be searched against a reference library in order to determine whether the group of ions represents a known entity or whether the group of ions represents a new or an unknown (a biochemical that is not in the library) chemical entity. If the group of ions is determined to represent an unknown chemical with no reference library entry, a new spectral entry is added to the library so that the unknown entity can be tracked in future studies. An attempt is made to assign chemical intelligence to the ions belonging to the unknown entity based on previously defined mass relationships, e.g. Na adduct m + 23.

## Conclusion

The QUICS method greatly accelerates the organization of ions into chemically related sets and expedites the creation of chemical library entries and the identification of metabolites. It is immensely beneficial to track both the chemicals for which there are authentic standard spectra in the chemical library and the chemicals for which there are no current library entries. Consequently new spectral libraries can be created automatically; the method is not limited by the availability of a chemical library of authentic standard spectra. Furthermore, the ability to access data across multiple samples provides a unique and powerful method to resolve co-eluting chemicals. Taken together these features greatly facilitate the chemo-centric approach to the analysis of metabolomics studies leading to the discovery of novel biomarkers and understanding of the underlying biochemical processes.

## Competing interests

CD, AE, HD and KL are employees of Metabolon, Inc.

## Authors' contributions

CD, AE and HD participated in the design and analysis of this study. CD, AE, HD and KL participated in manuscript preparation and read and approved the final manuscript.
